# You get what you screen for: on the value of fermentation characterization in high-throughput strain improvements in industrial settings

**DOI:** 10.1007/s10295-020-02295-3

**Published:** 2020-08-02

**Authors:** Maren Wehrs, Alexander de Beaumont-Felt, Alexi Goranov, Patrick Harrigan, Stefan de Kok, Sarah Lieder, Jim Vallandingham, Kristina Tyner

**Affiliations:** Zymergen Inc., 5980 Horton Street, Suite #105, Emeryville, CA 94608 USA

**Keywords:** Fermentation characterization, Microbial physiology, Strain optimization, Industrial bioprocess, High-throughput strain engineering

## Abstract

While design and high-throughput build approaches in biotechnology have increasingly gained attention over the past decade, approaches to test strain performance in high-throughput have received less discussion in the literature. Here, we describe how fermentation characterization can be used to improve the overall efficiency of high-throughput DBTAL (design-build-test-analyze-learn) cycles in an industrial context. Fermentation characterization comprises an in-depth study of strain performance in a bioreactor setting and involves semi-frequent sampling and analytical measurement of substrates, cell densities and viabilities, and (by)products. We describe how fermentation characterization can be used to (1) improve (high-throughput) strain design approaches; (2) enable the development of bench-scale fermentation processes compatible with a wide diversity of strains; and (3) inform the development of high-throughput plate-based strain testing procedures for improved performance at larger scales.

## Introduction

Industrial bioprocesses use microbial hosts to produce a variety of products, including commodity chemicals [1–3], specialty flavors and fragrances [4–6], and pharmaceutical ingredients [7, 8]. While most traditional bioproducts are native to the production microbe, such as primary and secondary metabolites [9, 10], the advent of recombinant DNA technology has enabled microbial production of heterologous molecules (not native to the host’s metabolism) [11]. Typically, extensive strain optimization and fermentation process development using laboratory bench-scale bioreactors (0.5–5 L) are required for successful and cost-effective production at industrial scales (e.g., 50–500 m^3^).

Historically, strain improvement approaches have been dominated by random mutagenesis or directed evolution, followed by high-throughput screening to identify novel or enhanced phenotypes in existing microbial hosts [12]. While this approach typically does not introduce novel enzymatic functionalities, it allows for strain improvement without an in-depth understanding of microbial metabolism and potentially searches the entire host genome for beneficial mutations. Typically, candidate strains that underwent random mutagenesis are first screened using a high-throughput method (e.g., 96-well plates) before being validated in a (lower throughput) bioreactor experiment that can more accurately reproduce industrial production conditions. The development of recombinant DNA technology in the 1980s paved the way for targeted genetic modifications of microbial production hosts, including gene knock-outs, knock-downs or overexpression of endogenous genes, as well as the introduction of heterologous genes with new enzymatic functionalities. However, early genetic engineering methods were labor intensive and low-throughput. As a result, genetic edits were rationally selected, with a focus on well-characterized genes and pathways likely to have positive effects on strain performance. Each individual strain could be tested directly in a bioreactor under conditions specifically optimized for its performance. This strategy led to impressive progress for several products, including 1,3-propanediol, insulin, and amino acid production [13–15]. However, rational strain engineering efforts are hampered by the contemporary, limited understanding of the microbial metabolism, and low success rates are likely for unstudied pathways and organisms. The emergence of “-omics” technologies (e.g., transcriptomics, proteomics, metabolomics, fluxomics) in the 1990s and the corresponding advances in metabolic modeling significantly increased the success rate of rational strain design [16–18]. However, despite these technological advances, the complexity of the microbial metabolism remains an obstacle during rational strain improvement attempts. As a result, the introduction of (rational) genetics edits derived from these large datasets and metabolic models does not always improve strain performance to the desired level.

Recent advances in DNA synthesis [19], DNA sequencing [20], genetic engineering [21–23], and automated strain engineering have enabled empirical strategies that address the limitations of rational strain engineering given the complexity of cellular metabolism. Rather than attempting to first understand the metabolic 'problem' before carefully designing a small set of targeted genetic ‘solutions’, high-throughput strain construction and testing strategies enable an empirical approach that starts with the ‘solutions' first. Once thousands of strains are designed, built, and tested, analyzing which of these genetic modifications affect performance can identify the areas of metabolism that are relevant to product formation. This circumvents the need to capture the complexity of microbial metabolism in a single comprehensive model.

While an empirical approach requires high-throughput strain engineering, increased throughput alone does not guarantee increased success. The large number of novel engineered strains introduces new technical challenges that must be carefully considered to correctly assess their performance. These include:

1. Efficient strain design. Even though building thousands of strains in a high-throughput, low-cost manner is feasible, rapid progress requires efficient strain design. This includes the prioritization of likely beneficial edits and the careful consideration of the genetic background that is used to identify favorable edits.
2. Flexible fermentation processes. With an increasing number of engineered strains, it becomes more likely to encounter strains that phenotypically differ significantly from their parent strain. As it is impractical to optimize the fermentation process for each individual strain, a generic fermentation process must be developed that can assess the potential of a wide diversity of strain phenotypes.
3. An efficient high-throughput test platform. The majority of engineered strains will only ever be tested in high-throughput microtiter plates. Thus, it is important that the strain performance during plate cultivations be predictive of the performance at production scale.

In this paper, we describe how the method of fermentation characterization can facilitate meeting the above demands and can be applied to improve the efficiency of a high-throughput strain improvement process. We first provide an overview of fermentation characterization and then describe its application to strain design, strain testing in bench-scale bioreactors, and strain testing in microtiter plates.

## Fermentation characterization

Strain performance validation experiments in bioreactors usually involve fairly minimal data collection. For example, one collects data at only few time points (beginning, middle, and final time points) on only a few analytical measurements (e.g., substrate usage, biomass, and product concentration) to evaluate the impact of the genetic edit on strain performance (e.g., titer, rate, or yield). In contrast, fermentation characterization comprises a much deeper analysis to gain a deeper understanding of the physiology of production strains within a given fermentation process. We seek to strike a balance between economical considerations and scientific value of bioreactor experiments aimed to validate strain performance, improve fermentation processes, and identify key drivers of strain performance.

During a fermentation characterization experiment, we run a standard fermentation that includes additional sampling time points around important phases in the fermentation process. In typical fermentation characterization experiments, we take samples at regular intervals, with a focus on important stages within the process. These include critical process times (beginning and end of the seed train and batch phase); times when there are changes in growth phases or viability; stages with observed metabolic shifts indicated by changes in oxygen requirements or pH profiles; and the onset of byproduct and/or product formation phases (Table 1). We then conduct a comprehensive data analysis to inform which aspects of the fermentation have the greatest impact on strain performance in a given process.

**Table 1:** A non-exhaustive list of measurements and standard calculations that may be performed on each sample during a fermentation characterization experiment

Goal	Measurement	Calculation	Description
Understand growth profiles	Biomass (OD, DCW, cytometry)	Growth rates	Calculation of maximum specific growth rates (*μ*_max_) during exponential phase, doubling time (*t*_d_), timing of exponential growth within the fermentation, number of cell doublings within the fermentation (both main fermentation alone and including entire seed train)
Understand production rates and profiles	Product concentration, cell concentration	Production rates	Calculation of the rate of product formation (*q*_p_), both over the course of the fermentation and during specific intervals
Quantify residual substrate and substrate uptake rates and profiles	Substrate concentration, cell concentration	Substrate consumption rates	Calculation of the rate of substrate consumption (*q*_s_), both over the course of the fermentation and during specific intervals
Understand production efficiency	Product and substrate concentration	Production yields Carbon balances Yield coefficients for different substrates	Calculation of the efficiency of product formation (*Y*_SP_ = *q*_p_/*q*_s_), both over the course of the fermentation and during specific intervals Account for all the carbon substrate added to the fermentation in biomass, product, pathway intermediates and byproducts (including CO_2_) Calculation of the mass of cells produced per unit mass of substrate consumed—*Y*_x/s_ during exponential growth
Identify and quantify pathway bottlenecks	Concentration of Pathway intermediates and byproducts	Byproduct formation	Calculation of the amounts of different byproducts/pathway intermediates formed during specific phases of the fermentation
Identify potentially limiting nutrients and substrates	Quantification of trace elements, Vitamins, AA, OA, Alcohols, N, P, S	Yield coefficients	Correlation of exhaustion of essential nutrients and decrease in growth rate or cessation of growth
Monitor cellular metabolism during fermentation	Offgas (CO_2_, O_2_ Ethanol)	Analysis of offgas and online measurements	Calculation of Oxygen Uptake Rates (OUR), Carbon Dioxide Evolution Rates (CER) and Respiratory Quotient (RQ) throughout the fermentation. Interpret OUR, CER, RQ, DO and pH trends in the context of identifying growth phases, metabolic shifts and loss of metabolic activity
Monitor the process and measure substrate uptake rates	Online measurements (pH, DO, feed rates, agitation, temperature)		
Quantify viability loss during fermentation	Viability (cytometry, CFUs)	Viability	Quantification of viability loss during fermentation
Monitor cellular morphology and broth viscosity during fermentation	Microscopy Viscosity	Morphology Viscosity	Documentation of cellular morphology and viscosity during the fermentation

*OD* optical density, *DCW* dry cell weight, *AA* amino acids, *OA* organic acids, *N* nitrogen, *P* phosphate, *S* sulfate, *DO* dissolved oxygen, *CFU* colony forming units)

These additional measurements, calculations, and accompanying interpretations enable a greater understanding of the fermentation process and provide insight into growth and production trends. For example, the product may be growth-associated, or may primarily be produced during stationary phase. We may observe the loss of viability at the end of the fermentation caused by a potential buildup of pathway intermediates or (by)products. Thus, an in-depth understanding of strain physiology within the fermentation process allows us to have a greater understanding of the factors that may impact strain performance. Together, the collection of these analyses and interpretations can help us both inform strain design strategies and create flexible and robust testing platforms that capture the characteristic phenotypes of a given process.

## Informing strain engineering approaches

Engineering a strain for improved performance in an industrial bioprocess requires optimizing over a genetic search space that is both large and complex. While the latest advances in genetic engineering facilitate building and testing thousands of strains with individual genetic edits, the number of strains needed to characterize the interactions between these edits increases factorially (Table 2). This situation is made more difficult by the fact that a strain’s phenotype is the result of a complex interaction between its genetics and its environment. As strain engineering and process development are often performed in parallel, the task becomes not only to build a variety of strains but also to assess their performance under a variety of process conditions. Clearly, this large search requires not only a high-throughput strain building platform but also a guiding framework that can help focus and prioritize strain engineering strategies. To this end, we propose the periodic, targeted characterization of strain physiology and performance during fermentation to increase the success rate of any genetic search for improved strains and processes. Factors that are of specific interest to strain design include the production of core pathway intermediates and byproducts, distribution of carbon flux, and the accumulation of potentially toxic products.

**Table 2:** Genetic search space size

Organism	Genome size (ORFs)	Strains needed to test all pairwise gene edits	Strains needed to test all sets of 10 gene edits
*Escherichia coli*	> 4400	> 10^5^	> 10^29^
*Saccharomyces cerevisiae*	> 5300	> 10^7^	> 10^30^
*Aspergillus niger*	> 14,000	> 10^7^	> 10^34^

Libraries of strains with individual edits targeting each gene/ORF in the respective genome contain ~ 10^3^ individual strains. Upon testing the interactions between genetic edits (multiple targets per strain) the library size increases by many orders of magnitude

These studies should focus first on the core pathway that produces the product of interest to identify any metabolic bottlenecks that may mask or hide the effect of beneficial off-pathway genetic edits. Identifying such bottlenecks requires time course measurements of both the end product and core pathway intermediates. If, during the fermentation process, a product plateau coincides with the accumulation of a pathway intermediate, the enzyme that uses this intermediate as a substrate becomes a target for strain engineering. Such a bottleneck can also be inferred from the accumulation of a byproduct formed from a pathway intermediate. In this situation, a limiting reaction drives flux towards an off-pathway reaction.

**Fig. 1:**
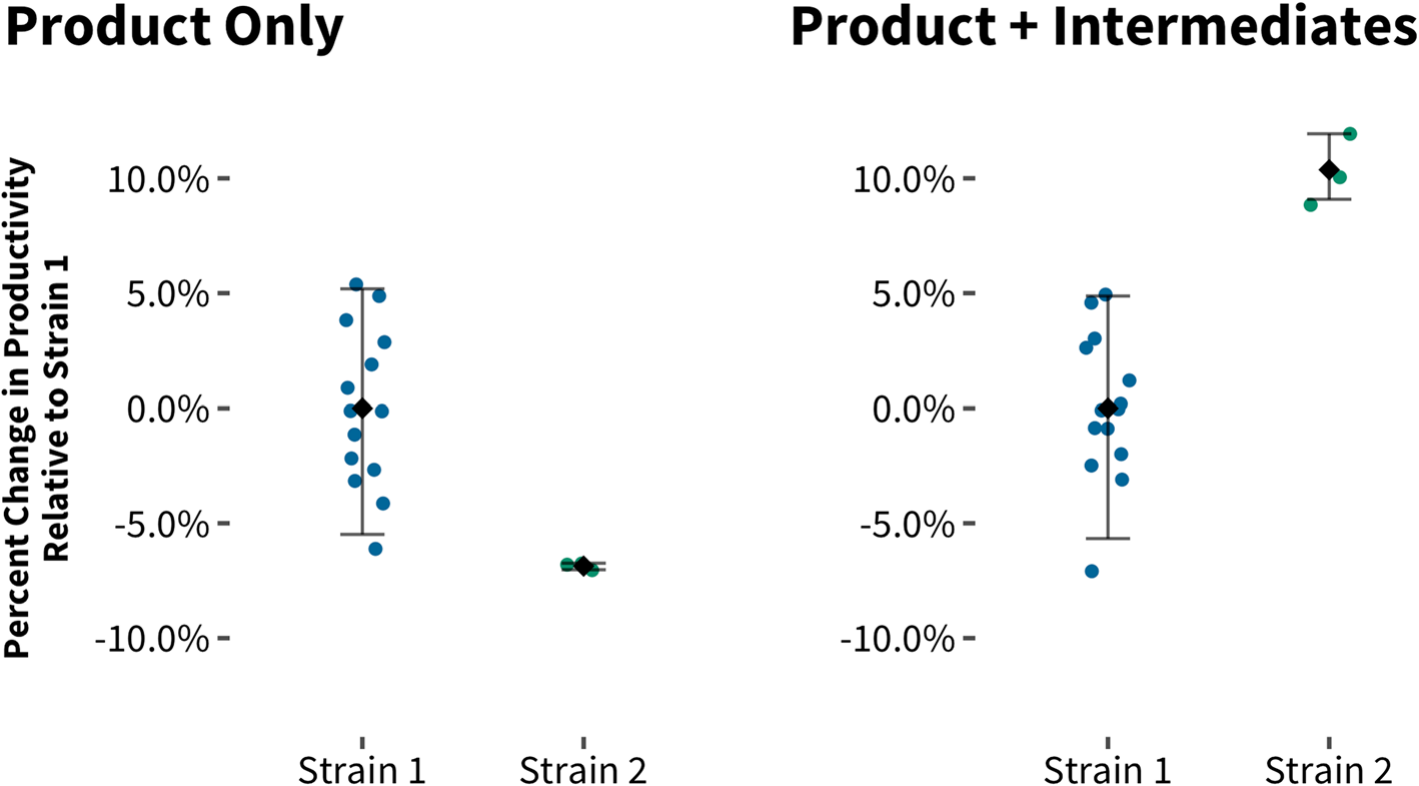
Systematic measurements of intermediates and final products of strains run in fermenters resulted in the identification of a strain (Strain 2) with a higher level of total pathway flux (i.e., product plus intermediates) compared to its parent strain (Strain 1). Limiting measurements to final product titers would have not allowed the identification of the improved total pathway flux of Strain 2 (Product only). Once a strain with improved total flux (Product plus Intermediates) is identified, subsequent overexpression of terminal pathway enzymes may result in a strain with improved (product) productivity. Note: As the precursor and product have different molecular weights, we performed a molecular weight correction to calculate how much product could be made out of the precursor

It is important to identify and eliminate pathway bottlenecks periodically, especially given that strain performance is often assayed via a single measurement of final product titer. In this situation, if a bottleneck exists in one enzyme in the pathway, then only edits that directly affect that enzyme will be identified as beneficial. Any potentially beneficial edits that drive more metabolic flux to the core pathway will be missed, as this flux will result in the accumulation of an unmeasured byproduct without improving final titer. In a previous project, we were tasked to improve the productivity of a production strain. A baseline fermentation characterization experiment, including pathway intermediate quantification, revealed that pathway intermediates were accumulating to a total level of ~ 10% of total pathway flux in the current production strain. This finding indicated the presence of a kinetic bottleneck in the pathway. As a result, any increase in upstream pathway flux will most likely result in a higher level of accumulation of pathway intermediates and not be detectable in final product formation. We then focused our strain engineering strategy to “debottleneck” the terminal pathway by increasing expression levels of the final pathway enzymes. Additionally, we systematically analyzed all historical strains for the sum of the pathway intermediate and final product. Using this modified screening strategy, we were able to identify a strain with a higher total pathway flux than the current top producer strain, although its product formation level was similar to the parent strain (Fig. 1). Overall, this adapted screening method helped us identify a wider range of improved strains and reduce the rate of false negatives. Failure to identify beneficial edits due to these types of epistatic interactions is likely a major driver of inefficiency in strain improvement cycles. Generally, identification of a metabolic bottleneck should be followed by investigating whether or not any previously tested strains have accumulated high levels of the corresponding intermediate (Fig. 1).

Even an optimized core pathway can be limited by metabolic flux, as cells will use substrate for processes other than product formation such as for growth and cellular maintenance. Once the core pathway is relatively optimized and bottlenecks have been eliminated (or at least identified), strain edits can be tested that divert carbon away from core metabolism or towards pathways that produce cofactors consumed in the core pathway. Unproductive carbon fluxes can be directly identified by measuring the accumulation of an unwanted metabolite, such as a soluble amino acid or a fermentative byproduct. In the absence of such measurements, a carbon mass balance can determine if a significant amount of carbon is unaccounted for, and to inform whether or not it is worth testing edits that generally alter metabolic fluxes [2].

**Fig. 2:**
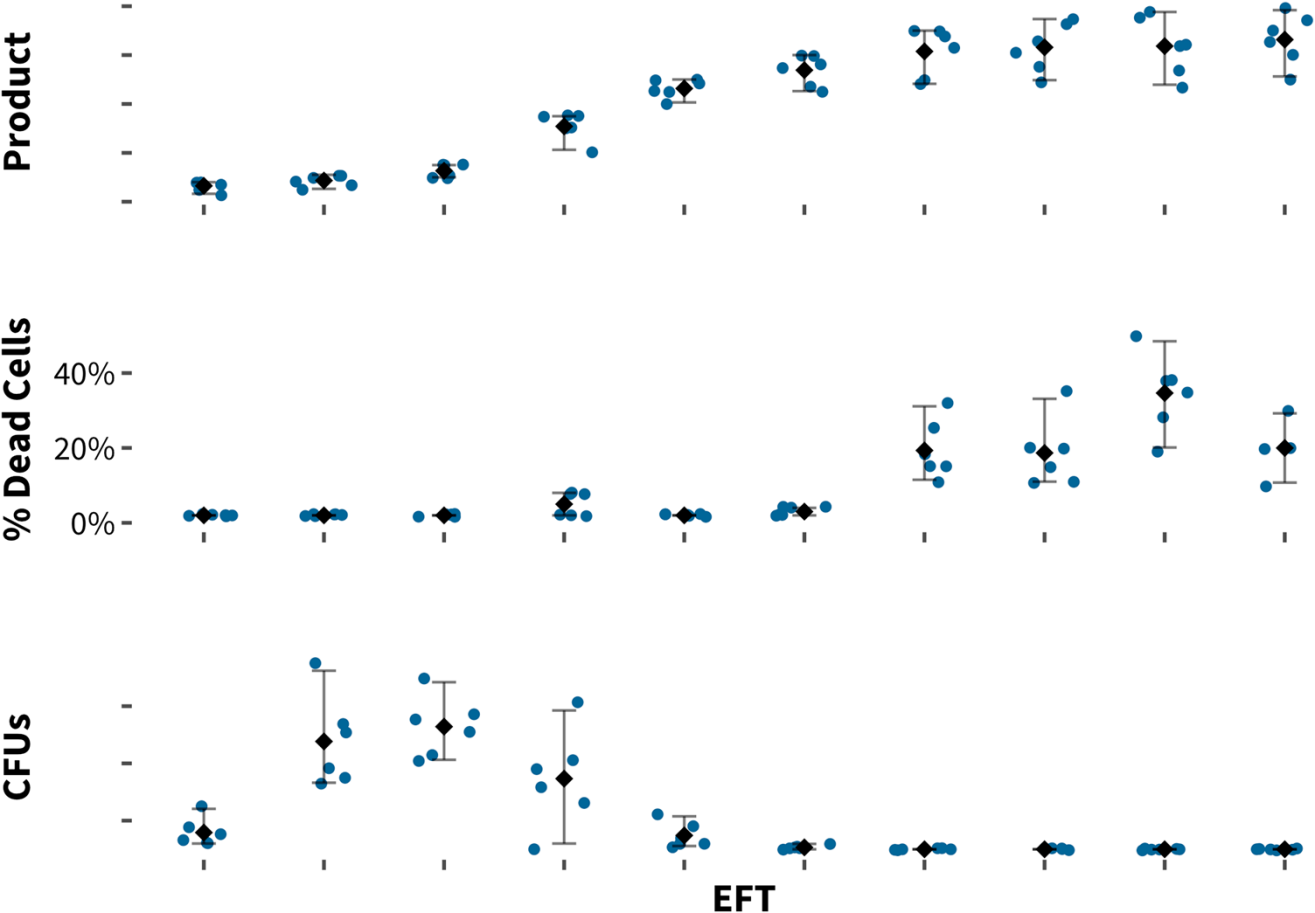
Plateau in product formation correlates with loss of viability and is indicated by an increase in the percentage of dead cells and a decrease in colony-forming units (CFU). We determined the percentage of dead cells by propidium iodide viability staining, followed by flow cytometry

In addition to characterizing product and byproducts formation, it is also important to track macro- and micronutrient accumulation, including but not limited to vitamins, trace elements, organic acids, alcohols, amino acids as well as carbon, phosphate, sulfate, and nitrogen sources. While nutrient limitation is often more easily addressed by changes to media formulation and the fermentation process, the accumulation of a product or byproduct to a potentially toxic level provides a target for genetic engineering. Such toxicity can be identified by a careful assessment of cell viability during the course of fermentation. While a plateau in productivity might be the result of a strain containing a pathway bottleneck, it might also be the result of cell death, as it is certainly true that dead cells don’t make products. In Fig. 2 (top panel), we assayed strains that showed a decreased productivity towards the end of fermentation for viability by counting colony-forming units (bottom panel) and staining with propidium iodide (middle panel). We found that the decrease in productivity coincided with a decrease in cell viability, leading to an investigation of the cause of cell death. Observations from a baseline fermentation characterization experiment helped to formulate hypotheses around the cause for the loss in viability. In this case, several causes seemed plausible, including product toxicity, the buildup of toxic pathway intermediates, and the consumption of the key nutrient in the fermentation broth. Once the root cause is identified, it is important to loop back to strain engineering efforts: if product toxicity is the root cause of loss of viability, then strain development efforts may include libraries of exporters. If the buildup of pathway intermediates is the root cause, strain engineering efforts may focus on improving flux in that part of the pathway.

Similar to pathway bottlenecks, inhibitory effects from product accumulation or media components might have masked beneficial edits in strains that were tested before this inhibition became known. Thus, it is important that repeated rounds of strain optimization also be followed by repeated rounds of strain characterization. Failing to identify phenotypes that might be masking edits that could improve strain improvement can lead to an increase in false negatives and directly impact the efficiency of an expensive high-throughput strain building platform.

## Enabling the development of bench-scale fermentation processes suitable for a wide variety of strains

High-throughput strain engineering platforms facilitate the introduction of a plethora of genome modifications targeted at strain performance improvements in a wide variety of host organisms. Zymergen works with clients to improve their specific production strains in processes to which they are already wedded. In these cases, we help mature production processes that are tuned to the client’s previous best strain; this, in turn, results in our dealing with fermentation of a wide strain diversity as we progress. We can also select microbial production hosts and develop scalable production processes in conjunction with strain optimization efforts to produce a variety of different products and materials in our internal projects. This involves developing fermentation processes from the ground up; we also need to work with a large strain diversity in these cases, though at a different stage of the fermentation characterization process. Thus, our ability to flexibly work with many different organisms at different project stages is essential to efficiently test strains for a multitude of different processes and products.

Once we have obtained our initial parent production strain, either from a client process or an internal product project, we apply both focused and genome-wide strain engineering strategies geared towards strain performance improvements to generate large quantities of engineered strains. Depending on the type of genome modification performed, the strains that emerge from our strain engineering pipeline may phenotypically differ significantly in many ways from its parent strain, including in growth rate, feed demands, and oxygen requirements. To accurately assess the optimal performance of strains from these diverse lineages and their respective optimal cultivation conditions, it is beneficial to develop fermentation protocols that can adapt to individual strain physiology.

With the large amount of strain diversity generated in Zymergen’s high-throughput build pipeline, it is important that we design fermentation processes with that in mind. When developing high-performing, bench-scale screening processes, we have two critical objectives: flexibility and robustness (defined as high process reproducibility and low statistical variability). For the former, the process must be flexible enough to accommodate wide ranges of phenotypic diversity and be able to react accordingly such that near-optimal strain performance is achieved. If a process is too rigid, in addition to harming our evaluation of a strain’s performance, an increase in variability may also be observed. Once we achieve a baseline process, we can apply fermentation characterization data to gain a deeper understanding of technical and biological parameters that are driving variability. By identifying these parameters, we are able to develop mitigation strategies to lower process variability. These, in turn, will directly impact our ability to identify improved strains while enabling the use of fewer fermentation runs to do so.

Many industrial fermentations are performed as fed-batch processes, in which a substrate-limited environment is maintained throughout the production phase to reduce overflow metabolism, drive metabolic flux towards product formation and reduce biomass accumulation. Typically, fed-batch fermentations start with a “main batch” phase, in which the cells metabolize the nutrients and carbon source provided in an initial growth medium, mainly to accumulate biomass.

**Fig. 3:**
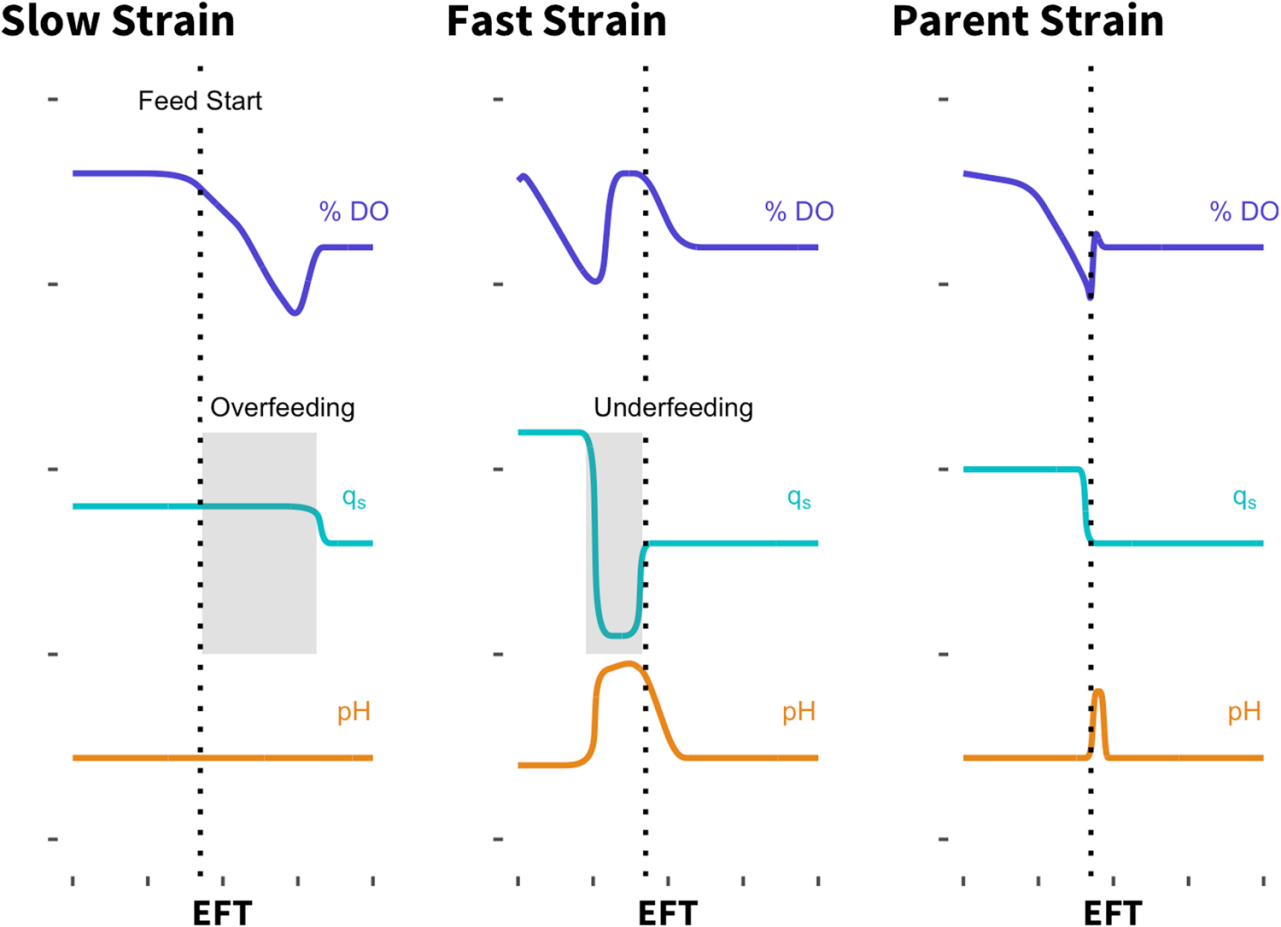
Impact of suboptimal feed initiation during a fed-batch process on strain physiology. We ran strains that exhibit different growth phenotypes compared to the parent strain in a fixed feed process that was optimized for the parent strain. For the faster-growing strain, the feed phase starts several hours after the cells have finished the batch phase, resulting in a phase of underfeeding or even starvation and decreasing the overall volumetric productivity by extending the fermentation by several hours. For the slower growing strain, the feed phase starts at a point where the cells have not completed the batch phase, resulting in overfeeding for several hours, likely negatively impacting product formation. qS is substrate uptake rate in grams of substrate per grams of cell per hour. This schematic assumes one-sided pH control (base addition only)

Typically, microbial cells undergo exponential growth when the carbon source is in excess and no limitations are exhibited (such as oxygen or other nutrients required for growth). This requirement is usually met during a batch phase in an aerobic fermentation process. Under these conditions, cells ideally grow at their maximal growth rate and double at their respective minimal doubling time. Once this “batch” phase in fed-batch processes is completed, the carbon source is delivered via an external feed to maintain cell viability and production. However, growth rates may differ significantly from strain to strain, leading to different time points of substrate exhaustion. As such, a fixed time initiation of feeds may result in starvation for fast-growing strains or overfeeding for slow-growing strains. Exhaustion of the carbon source in the initial growth medium is typically accompanied by various physiological signals, such as a rise in dissolved oxygen (DO) and potentially a pH spike (Fig. 3).

With an increase in actively growing cells, the oxygen demand of the culture increases which, in turn, results in a decrease in the dissolved oxygen present in the culture broth, assuming that the oxygen transfer into the culture is relatively constant. Once the carbon source in the initial growth media is depleted, the lack of substrate to be metabolized causes a decrease in the culture’s oxygen demand, resulting in a sudden spike in dissolved oxygen. Additionally, many organisms undergo overflow metabolism at their maximal growth rate, resulting in the formation of short-chain organic acids that lower the pH of the culture. Once the cells have consumed the initial carbon supplied in the batch media, they might switch their metabolism to allow consumption of organic acids, both produced during overflow metabolism or supplied in the media, which in turn results in an increase in pH. Thus, both DO and pH spikes indicate the full consumption of the main carbon source and can be exploited as triggers to initiate feeds.

The development of dynamic feeding protocols can present a means to account for differences in strain physiologies and help reveal conditions necessary for optimal strain performance by ensuring optimal carbon availability. Dynamic feeding protocols can include different aspects of the fed-batch process. On the one hand, they may include the automated start of the feed phase triggered by a biological signal that indicates that the cells have fully consumed the carbon source provided in the initial growth medium. On the other hand, these protocols allow dynamic control of the feed rate to account for different carbon and nutritional requirements during the fed-batch phase. The optimal rate at which the external feed is delivered to the culture is strain- and process-dependent. Deviation from the optimal feed rate to either a rate that is too low (underfeeding) or too high (overfeeding) can result in suboptimal strain performance that is not reflective of the true strain performance under optimal conditions.

**Fig. 4:**
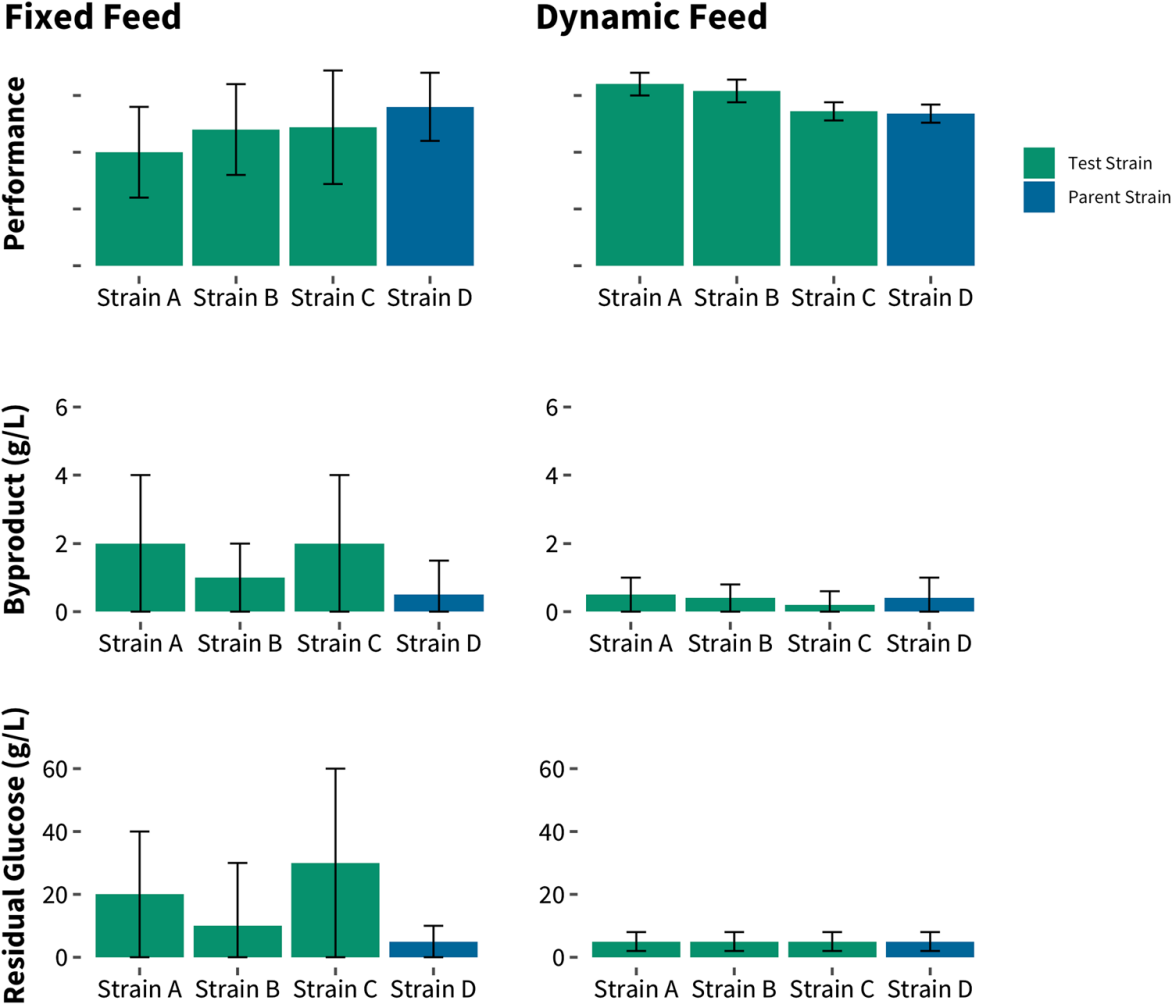
Strain performance is impacted by the feeding scheme. We ran strains in a fixed feed process that was optimized for a production strain. These strains produced large amounts of a detrimental byproduct, and we observed residual sugar at the end of the fermentation, indicating that the feed scheme did not result in optimal performance of these strains

An example of the potentially detrimental effect of screening strains under suboptimal feeding strategies is shown in Fig. 4. We ran several strains in a fixed feed process that was optimized for a production strain. The detection of residual glucose at the end of the run, as well as significantly increased byproduct formation for strains A, B, and C compared to the production strain, indicate that the selected feed rate was too high to reveal the true potential of these strains. Specifically, the production of byproducts can reduce yield and productivity, as certain byproducts can have an inhibitory effect on the critical genetic pathway or on growth. In contrast, underfeeding can reduce productivity and yield due to the relatively fixed carbon demands of biomass production and maintenance. In addition, suboptimal feeding strategies can result in increased variability in the performance of a single strain, as the feed rate cannot adjust based on subtle differences between replicates.

We can use the same physiological cues that can be exploited to initiate the fed-batch phase during the feeding phase to determine if the selected feed rate is suitable for strains that exhibit different growth or metabolic rates. There are various methodologies of feeding based on these biological signals, in which the pH or DO is controlled at a setpoint by adjusting the feed supply (pH stat or DO stat); a biological starvation signal, for example, a spike in DO or pH, may trigger a short feed pulse. Once the substrate supplied during this feed bolus is depleted, another biological starvation signal will trigger the next feed pulse. Other feeding strategies may be more complex. When implemented correctly, these dynamic feeding protocols can adapt to varying strain physiologies over the entire duration of the fermentation and thus consistently assess the optimal performance of each strain.

**Fig. 5:**
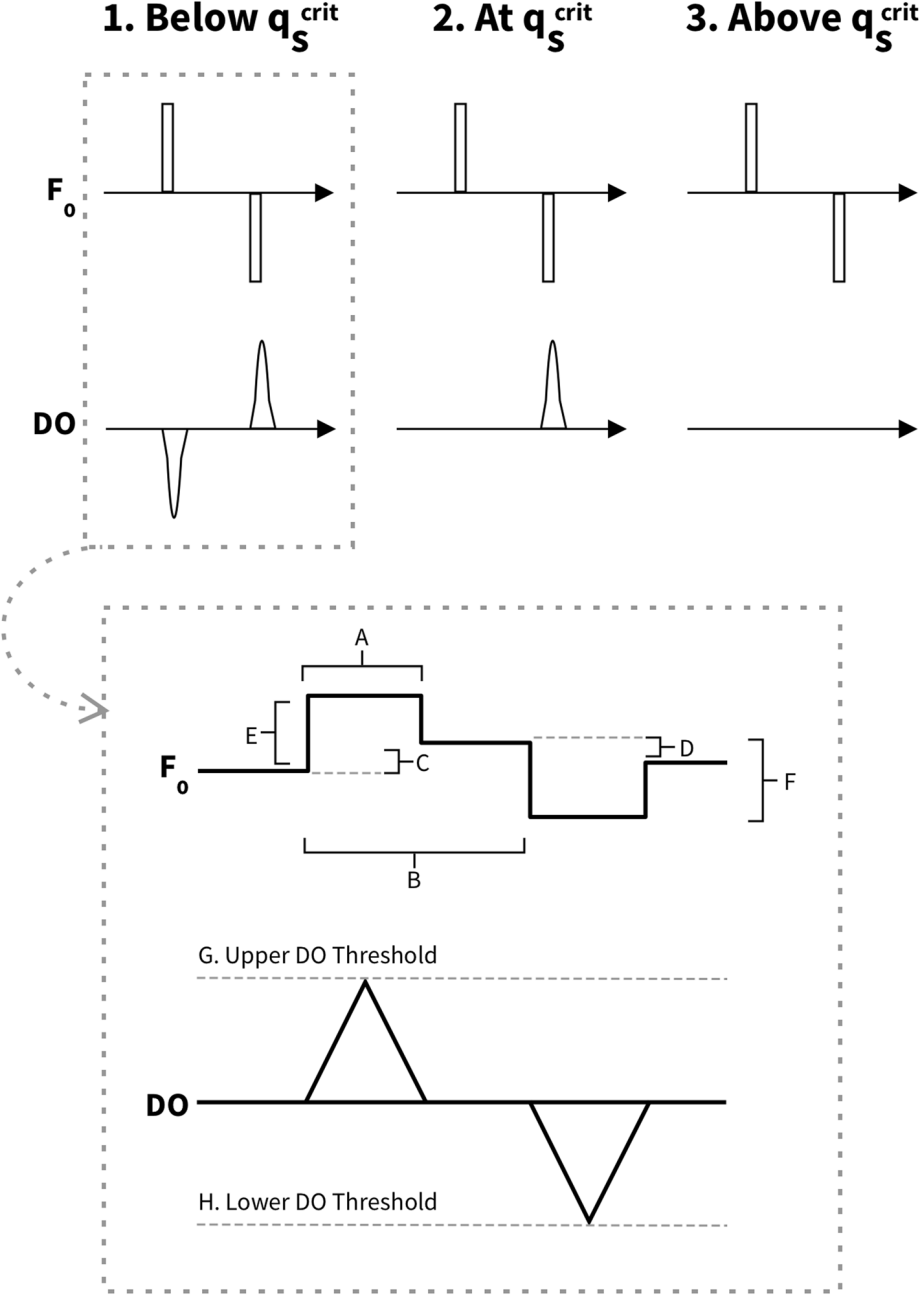
Development of a dynamic feed strategy, modified from Akesson et al*.* [24]. This strategy was developed using knowledge of the organism’s physiology, translating this to a process flow and optimizing different parameters including cycle length, pulse size, pulse duration, and magnitude of the feed rate change

Figure 5 shows an example of a dynamic feeding strategy that we implemented for an *E. coli* fermentation process, adapted from a strategy published by Akesson et al*.* [24]. To maximize product yield and productivity, the substrate feed rate must be optimized throughout the fermentation process to minimize byproduct production, mainly acetate in the case of *E. coli*. Acetate formation is a result of overflow metabolism and occurs as a result of overfeeding in aerobic cultures. A characteristic feature of overflow metabolism in *E. coli* is that the specific oxygen uptake rate reaches a maximum just prior to switching to overflow metabolism [24].

We used our knowledge of *E. coli* physiology to implement a probing strategy that adapts feed rates based on substrate demand throughout the fermentation (Fig. 5). The probing method tests the system periodically as it seeks to maintain the substrate feed rate, and thus the specific substrate uptake rate, just below the maximum oxygen uptake rate to prevent overflow metabolism. By periodically adjusting the feed rate up or down, and analysing the response of the dissolved oxygen concentration in the culture, we can determine if we need to decrease, increase, or maintain the current feed rate to reach an optimal substrate supply. We optimized several aspects of this strategy, including cycle length, pulse size, pulse duration, and magnitude of the feed rate change.

It should be noted that dynamic feeding protocols should take into account any given constraints of the process at scale, such as maximum feasible feed rates and oxygen transfer rates, to avoid screening strains in conditions that are not reflective of the production process at scale. Ultimately, we strive for optimal performance within the fundamental boundaries of the commercial-scale production environment to ensure that strain performance is preserved through scale-up. Once an improved strain has been identified using a dynamic feed protocol, it may be necessary to translate back to a fixed feed protocol that can be used at production scale and validate strain performance in a fixed feeding regimen.

In conclusion, employing dynamic feed initiation triggers and feeding protocols enables us to reveal the optimal performance of each strain and reduce variability. We translate the dynamic feeding profiles into fixed processes that can be run at a larger scale. This strategy allows us to efficiently assess optimal strain performance using dynamic screening processes and reliably scale our processes to production scale.

## Informing plate model development

To efficiently screen engineered production strains for improved performance at larger scale, we desire low-cost, high-throughput plate screens that are predictive of strain performance in bioreactors. While the technology to generate large numbers of engineered strains in a high-throughput manner has been successfully devised, the aspect of assessing strain performance across scales still presents a significant obstacle to identify optimized strains in small scale. As a result, we have set out to exploit fermentation data of processes performed at larger scales to inform our plate model development. However, generating these predictive plate models is challenging due to the inherent complexity of the fermentation processes of which they are designed to be predictive; developing successful models requires an in-depth understanding of the factors that impact strain performance during the production process.

We cannot control important process parameters known to affect strain performance, including substrate availability, pH, and DO, to the same extent in experiments in plate wells as we can in fermentors. For example, a typical industrial production process may have multiple seed stages to accumulate sufficient biomass for inoculation of the production tank, is subject to many controls (e.g., pH, DO), and may be performed in a substrate-limited manner (fed-batch). This allows for control over biomass accumulation during the production phase and ensures optimal metabolic flux towards product formation, aspects we cannot achieve in plate wells. Thus, the inherent limitations of performing cultivations in plates compared to in bioreactors that allow for a wide range of control and process capabilities will result in distinct differences in cultivation environments between these two platforms. In Table 3, we provide a summary of the main differences between cultivations in plates and tanks with known effects on strain physiology, and provide guidance on means to mitigate any potential detrimental effects on the predictability of the plate screen.

**Table 3:** Relevant challenges encountered during plate screen development and proposed potential solutions

Common challenges in plate screen	96-well plate	Lab-scale bioreactor	Details	Potential solutions
Population size (total number of cells)	10^7^ cells	10^12^ cells	Tank processes allow for an extended growth phase due to controlled environment	Match number of generations by adjusting the volume or density of the seed culture
pH control	Uncontrolled	Controlled base and/or acid addition	One-sided pH control is required for most fermentation process	Addition of buffering solutions, reduction of substrate/product concentration or biomass
Aeration	Uncontrolled	DO control through air/oxygen spargers and impeller speed adjustment	Oxygen transfer in plates is typically lower than in tanks	Characterize potential impact of oxygen limitation on strain performance. Reduction of oxygen uptake rate by adjustment of substrate concentration and cell density
Substrate load	Low	High	Strains may be sensitive to excess carbon and produce unwanted byproducts leading to changes in pH	Employ a glucose-limited main plate fermentation, either as batch or using glucose release system
Biomass concentration	Low	High	Oxygen transfer and process control capabilities in plates are limited	Include a scale-down factor in plate media composition
Carbon supply	Uncontrolled	Controlled	Bioreactors are equipped with controlled feeding mechanisms	Employ glucose release system and modify enzyme and substrate concentration to optimize release rate to generate fed-batch regimen
Evaporation	High risk	Low risk	Evaporation in plates can lead to significant differences in a culture volume	Determine the impact of evaporation on well-to-well CV at different time points. Minimize incubation time

The work-arounds to mitigate common differences between plate cultivation and fermentation in bioreactors presented in Table 3 alone are not sufficient to generate predictive plate models; the complex interaction of cells with their respective cultivation environment within this process will ultimately determine the final performance of a strain. Due to the lack of process controls in plates compared to bioreactors, it is critical to identify cultivation parameters that are most important to mimic in plates, and to understand which conditions can be more loosely defined without running the risk of compromising desired phenotypic characteristics. To put it succinctly, “You get what you screen for.”

**Fig. 6:**
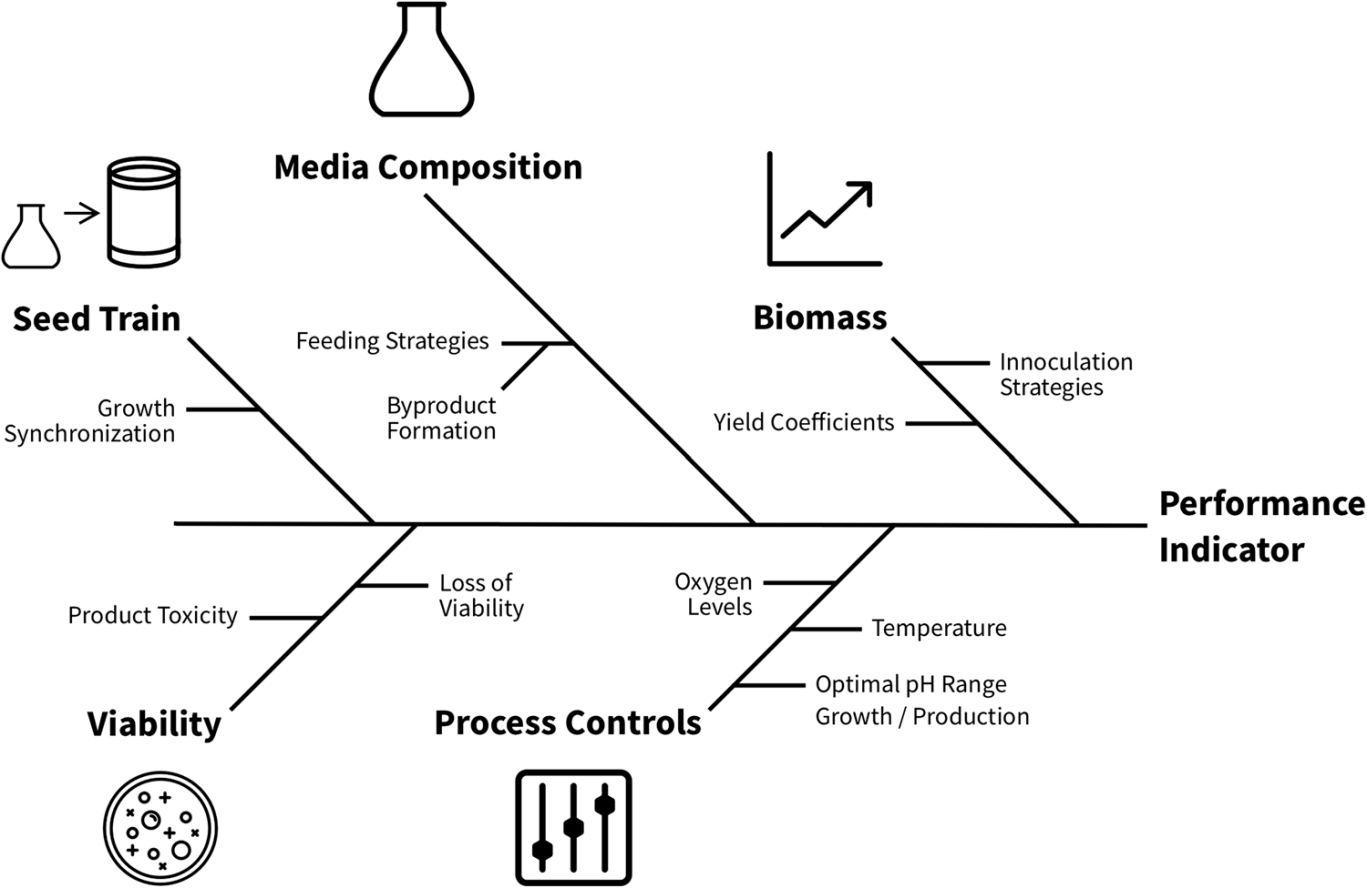
Basic knowledge of a fermentation process can inform baseline plate model cultivation conditions. However, there are many factors within a fermentation process, in addition to the protocol, that can impact performance KPIs (key performance indicators). These factors may be complex and can interact with each other to impact final KPIs

Knowing the protocols of an existing fermentation process, including media composition and main process controls, is an essential first step in designing a strain screening pipeline (Fig. 6). However, this is not sufficient for effectively developing predictive plate models for strain screening. Here, fermentation characterization data can be exploited to enhance our understanding of the impact of various cultivation parameters on strain performance of a specific process in bioreactors and to know which of these must be considered when moving from a highly controlled bioreactor environment into the plate environment for high-throughput strain screening. As such, a thorough fermentation characterization experiment is typically the first step on a quest to design a predictive plate screen.

We have identified various process factors and strain characteristics that can affect various strain performance indicators and have highlighted a selection of those in Fig. 6. Some of these factors can have significant complexity and interactions, such as oxygen demands and feeding strategies. In addition, the extent of the impact on strain performance for each of these aspects is highly dependent on the specific process and, thus, this exercise needs to be tailored to the individual production process. Having an understanding of which factors are most likely to impact yield, and how to screen for them, will help us develop a plate model that is predictive.

**Fig. 7:**
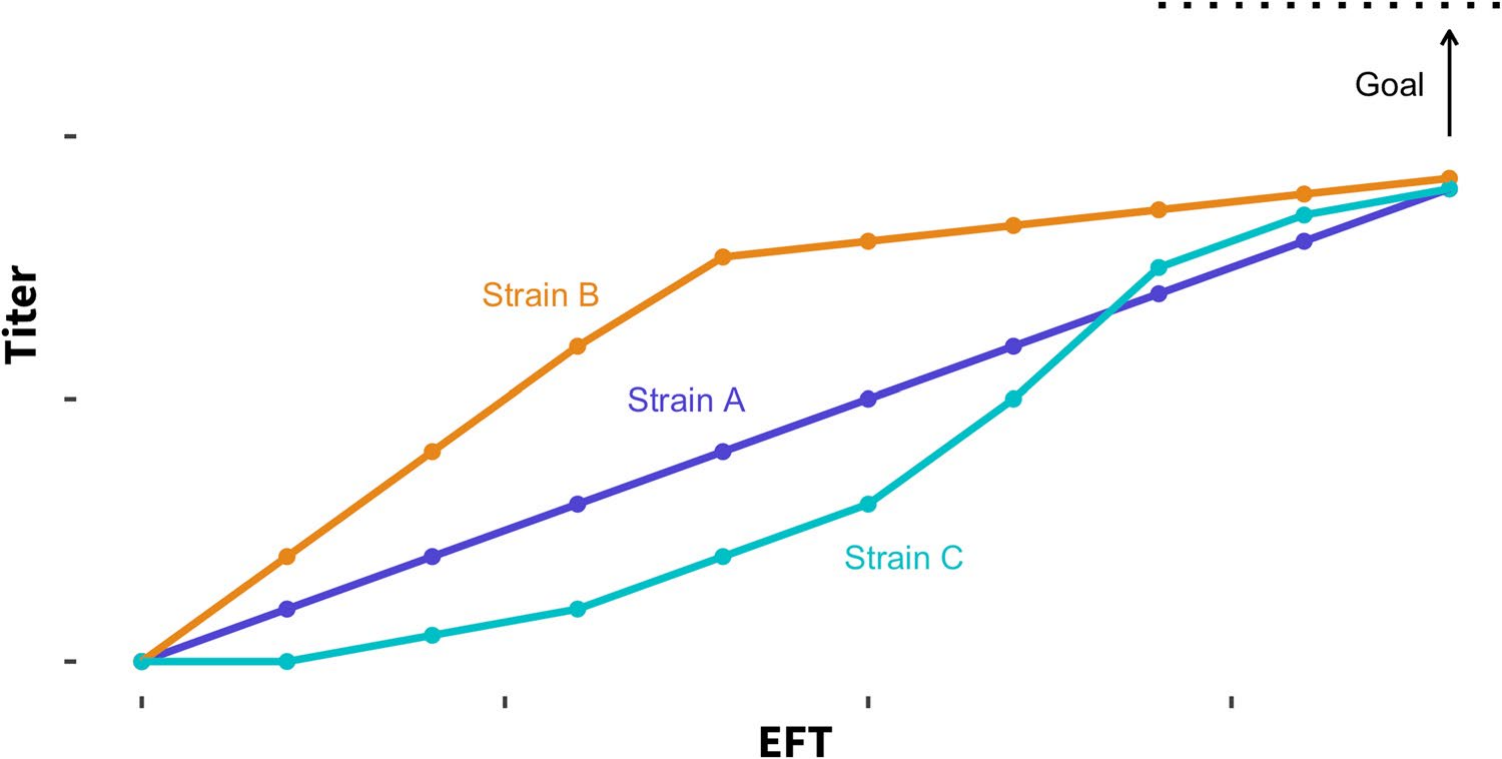
Final bench-scale fermentation results can be reached in different ways. An understanding of how final KPIs are reached in the fermentation process allows us to build predictive plate models

A typical strain performance indicator is productivity or the rate of product formation. For a productivity screen, the choice of screening strategy is highly dependent on how the final titer is reached and intermittent productivity intervals. Figure 7 shows three hypothetical ways to reach the same final titer within the fermentation process. Strain A displays a roughly linear accumulation of the product throughout the fermentation. In this case, a plate model would likely focus on increasing the linear rate of product formation. Means to increase the productivity of strains that exhibit this phenotype include optimization of metabolic flux through the pathway, engineering transporters and optimizing substrate uptake rates by metabolic engineering. Strain B accumulates the majority of the product in the first half of the fermentation. Diagnosis of the loss of productivity in the midpoint of the fermentation will be helpful in understanding the phenotype, designing plate models that help overcome this phenotype, and maintaining high production rates throughout the entire process. For example, the rapid decrease in productivity during the midpoint of the fermentation may be caused by product toxicity, decreasing strain viability. Screening for improved strains, therefore, may incorporate a plate model that screens for improved strain tolerance to the product. Strain C accumulates product slowly at the beginning of the fermentation. Again, diagnosis of the reason for the lag at the beginning of the fermentation will help to understand the phenotype and to design plate models to screen for improved strains. For example, the lag observed may be due to poor growth in the main batch media. Screening for strains that grow faster in this media may help improve early-stage (and overall) fermentation productivity.

**Fig. 8:**
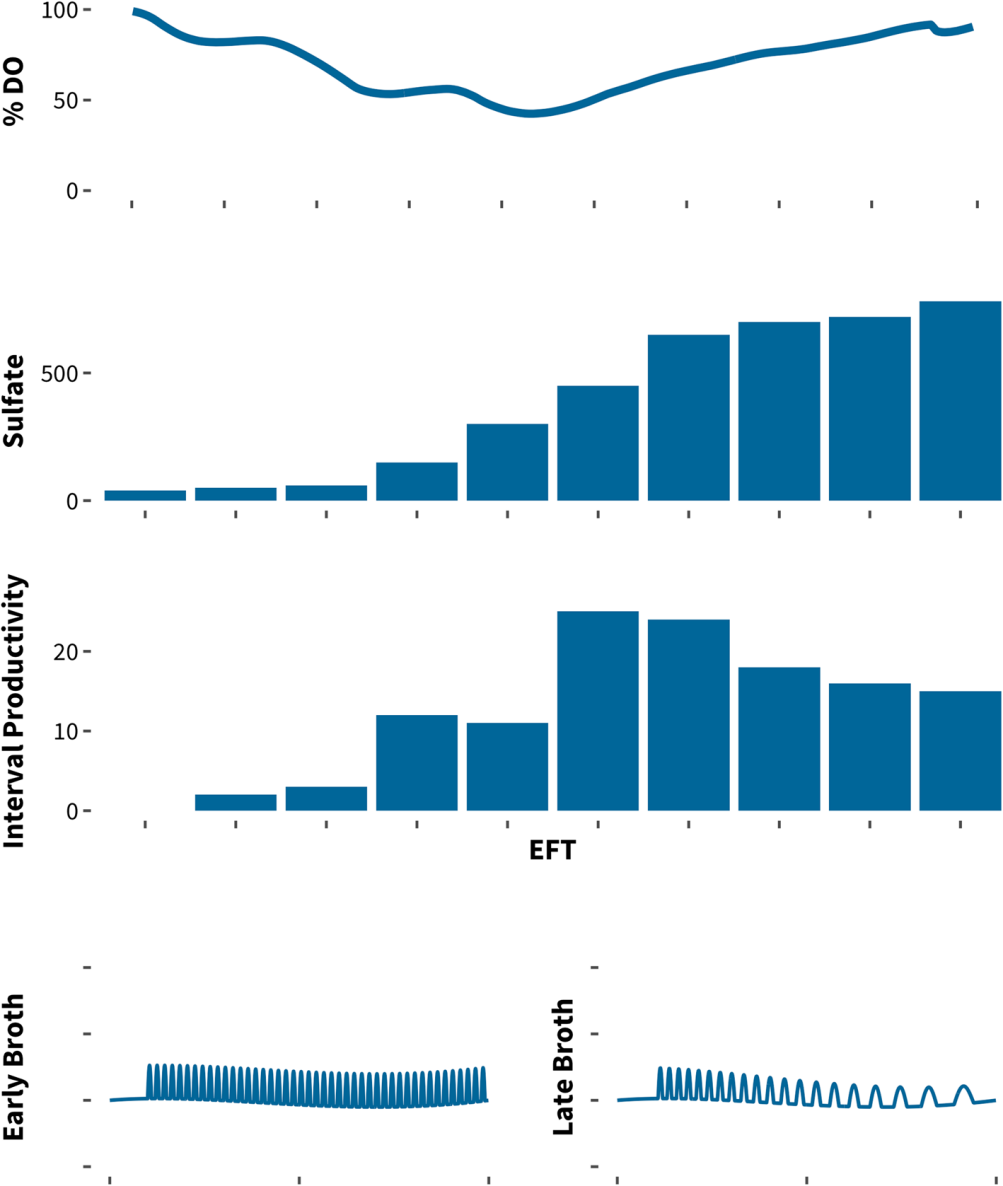
Peak productivities are observed in the middle part of the fermentation. High levels of residual sulfate, a byproduct of the primary nitrogen source, build up during the latter half of the fermentation coinciding with a decrease in interval productivity. A spent broth study confirmed the presence of an inhibitory factor, likely sulfate or the product itself in the fermentation broth, which would cause the observed decrease in productivity. The spent broth study was designed as follows: we harvested cells at peak productivity and inoculated them into the spent broth at early and late stages of the process. The dynamic feeding strategy employed in this process allows us to correlate feeding frequency with metabolic activity

A previous goal was to increase the yield of an amino acid production process, and thus to increase the amount product generated from a specific amount of substrate, without reducing productivity. Yield is often a valued aspect to improve when the cost of the substrate required for the process is high. As with many industrial fermentations, this process consisted of a biomass build phase during the initial stage of the main fermentation, followed by a production phase to limit biomass formation and efficiently divert carbon towards the product. To gain an enhanced understanding of this production process and inform our plate screen development and strain engineering strategies, we performed a fermentation characterization experiment that incorporated measurements of growth, product concentrations, cell viability, vitamins, organic acids, amino acids, and trace metals (Fig. 8).

We observed that peak volumetric productivities were being reached during the midpoint of the fermentation and that they decreased significantly at later stages of the process. Additionally, the timing of peak productivity and the start of declining productivity shifted towards an earlier time point for engineered high-yield strains compared to the initial production strain. In each case, the decrease in productivity in the midpoint of the fermentation coincided with a large buildup of residual sulfate in the broth, as a byproduct of the ammonium sulfate used as the primary nitrogen source. We observed no loss of cell viability.

At this point, our leading hypothesis was that the decreased productivity could be a result of the accumulation of high levels of sulfate. To test our hypothesis, we designed a spent media study in which we cultivated the production strain to the point of peak productivity as determined by our fermentation characterization experiment, harvested the cells by centrifugation, and then used these cells to inoculate spent broth from different fermentation stages.

In this process, external feeds are delivered dynamically in response to a biological response, and the frequency of delivery can serve as an indicator for metabolic activity of the culture. We observed that cultures inoculated into the spent broth from later stages of the fermentation process exhibited much lower frequencies of feed delivery, indicating lower metabolic activity compared to cells that were inoculated into spent media taken before the decrease in productivity. This observation suggested the presence of an inhibitory factor in the fermentation broth at later stages of the fermentation.

To implement new ideas for plate model development, it was essential that we take into account the various results of the fermentation characterization experiment, including the potential presence of an inhibitory factor in later stages of the fermentation, as well as the observation that higher-yielding strains experienced an earlier decline in productivity. We were able to formulate a new hypothesis that higher-yielding strains with equivalent productivity may result in higher levels of sulfate in the fermentation broth at earlier time points. To facilitate identifying strains with the ability to efficiently produce high levels of the target molecule even in the presence of high sulfate concentrations without a decrease in productivity, we included high levels of sulfate in the media in our plate models and significantly improved the predictive power of our plate screen. This example clearly demonstrates that observations from the fermentation can be leveraged to develop predictive plate models.

## Conclusions

We have provided insight into how we can leverage a deep understanding of the fermentation process and associated strain physiology to (1) inform strain engineering strategies to generate strains that show improved performance in a specific fermentation process; (2) develop robust and flexible fermentation processes that reveal the true strain performance of a given strain; and (3) inform the development of plate models that are predictive of performance across scales.

The advent of high-throughput strain engineering platforms has greatly increased the number of strains that can be constructed and has paved the way to a more empirical approach to address the challenge of identifying improved production strains. Now, thousands of potential solutions can be screened, and the associated diversity of strain performance leads to a new diversity of strain testing challenges. However, despite the high-throughput capabilities, prioritization of strain designs or “solutions” as well as adequate testing environments are critical to create efficient strain engineering and testing platforms. We have used our deep understanding of fermentation processes and microbial physiology to build an effective screening platform that allows us to identify the critical phenotypes that are predictive of strain performance so that these phenotypes can be modeled in high-throughput, small-scale screens.
